# Supplementary material for: Rapid turnover of life-cycle-related genes in the brown algae
Source: Genome Biol. 2019 Feb 14;20:35. doi: 10.1186/s13059-019-1630-6 (PMC6374913; doi:10.1186/s13059-019-1630-6)
Supplement: Supplementary file 3 — Protein alignments, corresponding Gblocks html file and CDS sequences for the lineage conserved gametophyte-biased genes. (ZIP 2109 kb) [file 13059_2019_1630_MOESM3_ESM.zip › Con_GA_alignments/Ec-21_003680.aa_ali.fasta-gb.htm]

Ec-21\_003680.aa.fasta.trans.aa\_ali.fasta


## Gblocks 0.91b Results

Processed file: **Ec-21\_003680.aa.fasta.trans.aa\_ali.fasta**  
Number of sequences: **2**  
Alignment assumed to be: **Protein**  
New number of positions: **702** (selected positions are underlined in blue)

```
                         10        20        30        40        50        60
                 =========+=========+=========+=========+=========+=========+
Ec-21_003680     MEEYEPIKVLGEGSFGKVYLMKHSKTRDLVCTKVIKLKNIPPKEQEACKNEVELLSRMCH
MSTRG.30424.1    MEEYEPIKVLGEGSFGKVYLMKHSKTRDLVCTKVIKLKNIPPKEQEACKNEVELLSRMCH
                 ############################################################


                         70        80        90       100       110       120
                 =========+=========+=========+=========+=========+=========+
Ec-21_003680     PNIVGYTNSFLYKNCLCIIMEYCDAGDLGDRVNEAKGQLLPESKVMTWFVQTALGLHFMH
MSTRG.30424.1    PNIVGYTNSFIYKNCLCIIMEYCDAGDLGDRVNEAKGQLLPESKVMTWFVQTALGLHFMH
                 ############################################################


                        130       140       150       160       170       180
                 =========+=========+=========+=========+=========+=========+
Ec-21_003680     SNRVLHRDIKTQNVFILSSGRVVLGDLGISKLLGGTRDFASTCIGTPYYMSPEIFKNHPY
MSTRG.30424.1    TNRVLHRDIKTQNVFILSSGRVVLGDLGISKLLGGTRDFASTCIGTPYYMSPEIFKNHPY
                 ############################################################


                        190       200       210       220       230       240
                 =========+=========+=========+=========+=========+=========+
Ec-21_003680     NDKSDVWALGCLLYELLTLKHAFDAQSLNGLAGKIIKGKFPSVSTQYSKNLRALVNDMLA
MSTRG.30424.1    NDKSDVWALGCLLYELLTLKHAFDAQSLNGLAGKIIKGKFPSVSSQYSKNLRALVNDMLA
                 ############################################################


                        250       260       270       280       290       300
                 =========+=========+=========+=========+=========+=========+
Ec-21_003680     TNPKKRPDIEQILRKSFVQTRVKEFVGDLDADELHRQEKAMHALSDQLIDLKMDDAVPMA
MSTRG.30424.1    TNPKKRPDIEQILRKSFVQTRVKEFVSDLDADELHRQDKAMHALSDQLIDLKMDDAVPME
                 ############################################################


                        310       320       330       340       350       360
                 =========+=========+=========+=========+=========+=========+
Ec-21_003680     NLIVEEGEGD-GPNAPSPSESDAAAAAAAAAA---KSGGSRSQLKAQKHQLELEEEKRIA
MSTRG.30424.1    NLIVEEGNGDEGPNAPSPSESDATAAAAAAAAASSKSGGSRSQLKAQKHQLELEEEKRIA
                 ########## #####################   #########################


                        370       380       390       400       410       420
                 =========+=========+=========+=========+=========+=========+
Ec-21_003680     AEMALNRLRRKAAERRRRASLSRSNSPMGGEGG--RIGS---GSPRYPGSPGSP---GSP
MSTRG.30424.1    AEMALNRLRRKAAERRRRASLSRSNSPMGGEGGGGRIGSGGGGSPRYPGSPGSPGAPGSP
                 #################################         ############   ###


                        430       440       450       460       470       480
                 =========+=========+=========+=========+=========+=========+
Ec-21_003680     DNGDTGTGG-GGGDGDGSHTRGRRPSILQRLGSLATSPLQSRRKSLSTPASPSGGNDS--
MSTRG.30424.1    ENGAGETGGSGGGTGDGSPSRGRRPSLLQRLGSLATSPIQSRRKSLSTPASPSGNNDSDS
                 ######### ################################################  


                        490       500       510       520       530       540
                 =========+=========+=========+=========+=========+=========+
Ec-21_003680     PRGG---SNNGSQSAR--GPGAGMSDVDKAMALAVAAAEKLDGDGDGDGGGGSAVAKGPT
MSTRG.30424.1    IRGSSGNKNNGSQSARAAGAGAGMSDVDKAMALAVAAAEKLEDGGGGGGDGGGVVTKGPT
                                   ##########################################


                        550       560       570       580       590       600
                 =========+=========+=========+=========+=========+=========+
Ec-21_003680     GAPRQSLGRPKEATDYVVNPTRRRSK----SGTNSNNSSGG--GEVSSRS-ARGSTG---
MSTRG.30424.1    GPPRQSLARPKEATDYVSNPTRRRSKGGVNSSSNSNNASGGSNGNISARSSTRGSSGGGG
                 ##########################    ###########                   


                        610       620       630       640       650       660
                 =========+=========+=========+=========+=========+=========+
Ec-21_003680     GGSPRGGGEN--GRPRASQDGESS--SKCHLSPDLPKLQLGANASATPALRSGRSSADRG
MSTRG.30424.1    GGSPREGRGEGHDSSRKSQEGESSSSSPCRPSPDLPKLQLGSNASATPALRSGRSSADRS
                                           ################################# 


                        670       680       690       700       710       720
                 =========+=========+=========+=========+=========+=========+
Ec-21_003680     GSPREGRGSGKGDSLFEEEDDSNDLFRVLKEQAKAHAKEGEDKEIHDREEELRAKLREAE
MSTRG.30424.1    N--------PKSEHLFEE-DDSNDLFHVLKEQAKAHAREGEDKEVHEREEELRAKLREAE
                                    #########################################


                        730       740       750       760       770       780
                 =========+=========+=========+=========+=========+=========+
Ec-21_003680     QRCLDLQSNMEKVREAGKRRGSIIVPPPPSGASTGGGEALDDMGLPKLSVDVNPSTRGSL
MSTRG.30424.1    QRCHELQESMEKVREAGKRRGSIAVAPP--AAPAGPT-----EAPPKLNIDVNPSTKGSL
                 ############################                 ###############


                        790       800       810       820       830       840
                 =========+=========+=========+=========+=========+=========+
Ec-21_003680     S-PGGGGSAQ--RYSRSVSNCSGDEDRASDFEDEPVPYCQSHLEEIHDAPTPVGNLVDRV
MSTRG.30424.1    SAPGGSGGTPRQSYSRSASSCSADDDRASDFEDEPVPYCQSHLEEIHDAPTPVGNLVX--
                 #            ############################################   


                        850       860       870       880       890       900
                 =========+=========+=========+=========+=========+=========+
Ec-21_003680     QFITKKCSDGLGEAKFAKALDLLKKMQEGDCMVTLDGVEYDGNEEDDVEKVLVKVIGKHK
MSTRG.30424.1    ------------------------------------------------------------
                                                                             


                        910
                 =========+====
Ec-21_003680     LHYWNLLDQLLLMS
MSTRG.30424.1    --------------
```

```
Parameters used
Minimum Number Of Sequences For A Conserved Position: 2
Minimum Number Of Sequences For A Flanking Position: 2
Maximum Number Of Contiguous Nonconserved Positions: 8
Minimum Length Of A Block: 10
Allowed Gap Positions: None
Use Similarity Matrices: Yes
```

```
Flank positions of the 12 selected block(s)
Flanks: [1  310]  [312  332]  [336  393]  [403  414]  [418  429]  [431  478]  [499  566]  [571  581]  [627  659]  [680  748]  [766  781]  [794  837]  

New number of positions in Ec-21_003680.aa.fasta.trans.aa_ali.fasta-gb:  702  (76% of the original 914 positions)
```
